# Gestational diabetes mellitus increases the detection rate and the number of oral bacteria in pregnant women

**DOI:** 10.1097/MD.0000000000014903

**Published:** 2019-03-15

**Authors:** Han Yao, Dan Xu, Zhichao Zhu, Guoyun Wang

**Affiliations:** Department of Stomatology, the Third Affiliated Hospital of Soochow University, Changzhou, Jiangsu, China.

**Keywords:** GDM, oral health condition, oral microbial imbalance, PSM

## Abstract

In the present study, we aimed to investigate the association between gestational diabetes mellitus (GDM) and the oral microbial imbalance in the second trimester of pregnancy.

Three hundred thirtyone women in the second trimester of pregnancy who underwent prenatal examinations at the Third Affiliated Hospital of Soochow University from February 2018 to August 2018 were included in this study. Personal parameters including the age, education level, and body mass index (BMI) at 28 weeks of gestation were recorded. Gestational diabetes mellitus (GDM) was diagnosed according to the Standards of Medical Care in Diabetes-2011 recommended by American Diabetes Association (ADA). After the significant difference of each parameter was erased by a propensity-score matched (PSM) analysis at a 1:1 ratio, the oral health conditions and the oral flora in pregnant women with GDM were compared with those in nondiabetic pregnant women.

Our data demonstrated that 65 of the 331 pregnant women (19.6%) were diagnosed with GDM. Results from the matched data including 59 matched pairs of pregnant women showed that the gingival index (GI), plaque index (PI), tooth mobility degree (TMD), probing depth (PD), and bleeding on probing (BOP) of pregnant women with GDM were higher or more severe than those of nondiabetic pregnant women (*P* < .05). The detection rate of tuberculosis bacilli, Black-pigmented bacteria, and Capnocytophaga in pregnant women with GDM was higher than those in nondiabetic pregnant women (*P* = .000, *P* = .026, and *P* = .030, respectively). In addition, pregnant women with GDM had fewer oral streptococci (*P* = .000) and lactobacilli (*P* = .000) and more oral anaerobic bacteria (*P* = .000), tuberculosis bacilli (*P* = .000), Black-pigmented bacteria (*P* = .007), Capnocytophaga (*P* = .000), and actinomycetes (*P* = .000).

The detection rate and the number of oral bacteria in pregnant women with GDM were higher than those in nondiabetic pregnant women in the second trimester of pregnancy.

## 1 Introduction

Gestational periodontitis and gestational diabetes mellitus (GDM) are 2 common complications in pregnant women.^[1]^ GDM is defined as diabetes or any degree of glucose intolerance that occurs during pregnancy in a woman who has no diabetes or glucose intolerance before pregnancy.^[2]^ In China, the incidence of GDM is 12.8% to 16.7% with a tendency of increasing year by year.^[3]^ GDM significantly increases the perinatal maternal and infant risks, including macrosomia, stillbirth, neonatal hypoglycemia, and increased cesarean section rate.^[4]^

There are a large number of microbes in the human mouth. Under normal circumstances, various microorganisms and their metabolites form an organic dynamic balance with saliva on the surface of specific tissues in the oral cavity, which is called oral microbial balance.^[5]^ Oral microbial balance plays an important role in resisting infection and maintaining normal morphology and function in various tissues of the oral cavity.^[5]^ Once this balance is broken, the normal flora may become an opportunistic pathogen, and foreign bacteria and pathogenic bacteria are more likely to colonize and reproduce in the mouth, eventually leading to oral infection.^[6]^ Previous studies have shown that if oral infection cannot be effectively controlled, some bacteria in the periodontal tissue may travel via the bloodstream toward the uterus, which can induce an inflammatory reaction, and ultimately lead to premature birth.^[7,8]^

Periodontitis is a common oral infectious disease that is often accompanied by oral microbial imbalance.^[9]^ Several studies have shown an association between GDM and periodontitis.^[10,11]^ However, the association between GDM and the oral microbial imbalance remains indefinitely. Therefore, we designed and conducted this observational study to investigate whether GDM correlates with oral microbial imbalance. Specifically, in the present study, the detection rate and the number of oral bacteria in pregnant women with GDM were compared with those in nondiabetic pregnant women in the second trimester of pregnancy.

## 2 Materials and methods

### 2.1 Subjects

Approved by the Ethics Committee of Soochow University, subjects were from pregnant women in the second trimester of pregnancy who underwent prenatal examinations at the Third Affiliated Hospital of Soochow University from February 2018 to August 2018. A total of 375 pregnant women were randomly selected to verify the gestational age and previous medical history. Only the pregnant women meeting the following criteria were eligible for inclusion:

1. women aged 14–28 weeks of pregnancy;
2. no family history of diabetes, no history of diabetes before pregnancy.

Several pregnant women were excluded according to the following criteria:

1. taking drugs that affect blood glucose (BG) levels (except estrogens);
2. having a history of diabetes or family history of diabetes;
3. having bad habits such as smoking and drinking;
4. having a history of severe chronic diseases such as liver disease, hypertension, malignant tumors, allergic diseases, infections, obesity;
5. have hyperemesis gravidarum, trophoblastic disease, pre-eclampsia.

Finally, 331 pregnant women were included in this study, and their age, education level, and body mass index (BMI) at 28 weeks of gestation were recorded. All included pregnant women have understood and signed the informed consent form.

### 2.2 Diagnosis of GDM

All pregnant women enrolled in the study underwent a 75 g oral glucose tolerance test (OGTT) at 28 weeks of gestation. Once GDM has been diagnosed by OGTT, glycated hemoglobin (HbA1c) was measured by high pressure liquid phase method. The diagnosis of GDM was performed according to the Standards of Medical Care in Diabetes-2011 recommended by American Diabetes Association (ADA),^[12]^ as listed in Table 1.

**Table 1:**
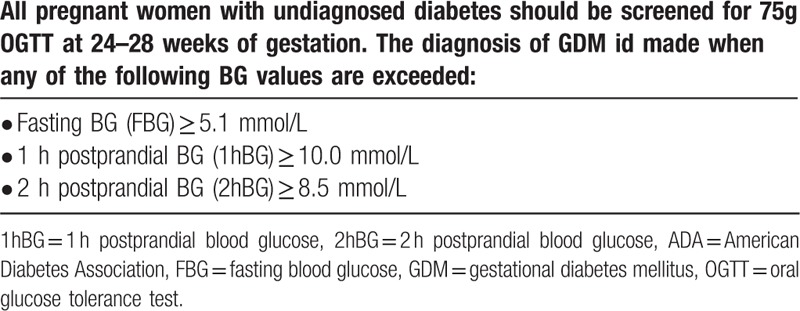
Standards of Medical Care in Diabetes-2011 recommended by ADA.

### 2.3 Investigating the oral health conditions

One eligible tooth of each subject was selected as the test tooth. The gingival index (GI), plaque index (PI), tooth mobility degree (TMD), probing depth (PD), and bleeding on probing (BOP) were observe.

### 2.4 Detecting the oral flora

The supragingival plaque of the subject's test teeth was wiped with a dry cotton ball. Subsequently, we collected the subgingival plaque at the bottom of gingival sulcus with a 1 mm diameter sterile stainless-steel ring until a thin film was formed on the surface of the ring. Then the ring was cut with a sterile scissors and placed in a bottle of 1 ml of physiological saline.

The sample bottle was shaken on a mixer for 30 seconds, and 0.1 ml of the solution was inoculated on a selective medium such as CDC-specific anaerobic medium, carbon dioxide phage, or actinomycetes. Anaerobic bacteria were cultured for 5 days and microaerobic bacteria were cultured for 2 days. Colony identification was performed using a VITEK automatic microbiological assay instrument, and colony count (CFU/ml) was performed.

### 2.5 Statistical analysis

The difference of measurement data was compared with *t* test. The difference of count data was compared with the Chi-Squared test (or Fisher exact test, if appropriate). The correlation between HbA1c and BG value was evaluated by Pearson’ correlation analysis. To adjust for differences between nondiabetic pregnant women and pregnant women with GDM, we performed a propensity-score matched (PSM) analysis at a 1:1 ratio. The PSM model was based upon age, education level, and BMI at 28 weeks of gestation. The difference of each variable was considered significant if two-sided *P*-values less than .05. Statistical analyses were performed using SPSS statistical software package, version 22.0 (SPSS Inc., Chicago, IL).

## 3 Results

### 3.1 Incidence of GDM in the second trimester

According to the diagnostic criteria of the 2011 ADA guidelines, 61 of the 331 pregnant women had a fasting BG (FBG) value greater than or equal to 5.1 mmol/L. 25 of the 331 pregnant women had a 1 hour postprandial BG (1hBG) value greater than or equal to 10.0 mmol/L. Nineteen of the 331 pregnant women had a 2 hours postprandial BG (2hBG) value greater than or equal to 8.5 mmol/L. Total 65 of the 331 pregnant women (19.6%) were diagnosed with GDM. Between nondiabetic pregnant women and pregnant women with GDM, the differences in FBG, 1hBG, and 2hBG value were significant (*P* < .05; Table 2).

**Table 2:**
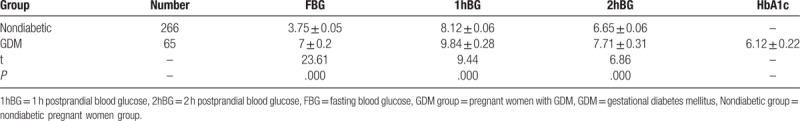
The differences in FBG, 1hBG, and 2hBG value between nondiabetic pregnant women and pregnant women with GDM.

**Figure 1:**
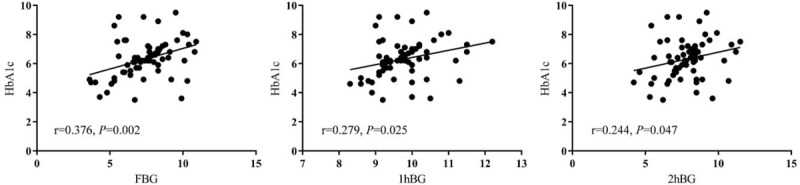
The correlation between HbA1c and each BG value in 75 g OGTT. Pearson’ correlation analysis was used to evaluate the correlation between HbA1c and FBG, 1hBG, and 2hBG in OGTT. HbA1c, glycated hemoglobin; 1hBG = 1 hour postprandial blood glucose, 2hBG = 2 hours postprandial blood glucose, BG = blood glucose, FBG = fasting blood glucose, OGTT = oral glucose tolerance test.

We used the Pearson’ correlation analysis to evaluate the correlation between HbA1c and each BG value in 75 g OGTT. The results showed that HbA1c was significantly correlated with FBG (*r* = 0.376, *P* = .002), 1hBG (*r* = 0.279, *P* = .025), and 2hBG (*r* = 0.244, *P* = .047) (Fig. 1).

### 3.2 Personal parameters of subjects

Table 3 shows the personal parameters of the two groups of pregnant women. The age of the women is categorized according to ages 18 to 24, 25 to 29, 30 to 35, and 35 to 44. The degree of education is categorized according to high school, polytechnic school, junior college, and bachelor degree or above. The BMI at 28 weeks of gestation was grouped according to 18.5 to 23.9, 24 to 27.9, and ≥28. There was a statistically significant difference in age and BMI at 28 weeks of gestation between the 2 groups (*P* = .002, *P* = .000, respectively).

**Table 3:**
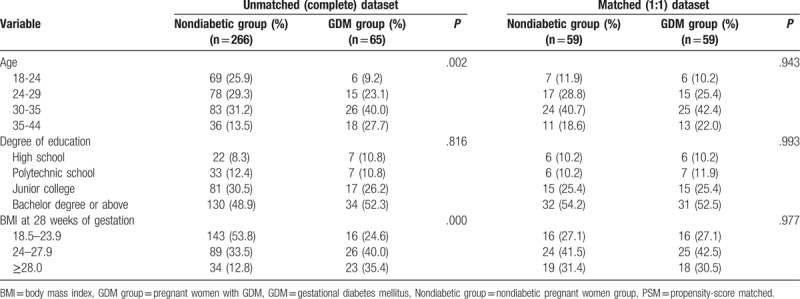
The clinical parameters of PSM cohorts for nondiabetic pregnant women and pregnant women with GDM.

To erase significant difference of each parameter, we performed a PSM analysis at a 1:1 ratio. The 1:1 matching for nondiabetic pregnant women vs pregnant women with GDM resulted in 59 matched pairs and a sample size of 118 pregnant women (Table 3). In the matched data, there was no significant difference in age, education, and BMI at 28 weeks of gestation between the 2 groups of pregnant women (*P* > .05).

### 3.3 Comparison of oral health conditions

We compared the oral health conditions of the matched two groups, as shown in Table 4. The GI, PI, TMD, PD, and BOP of pregnant women with GDM were higher or more severe than those of nondiabetic pregnant women (*P* < .05).

**Table 4:**
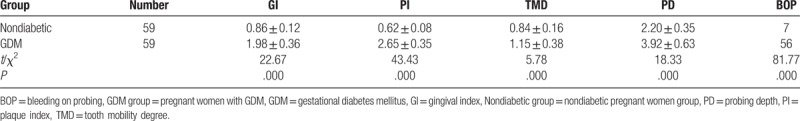
The comparison of oral health conditions between nondiabetic pregnant women and pregnant women with GDM.

### 3.4 Comparison of oral bacteria detection rates

We compared the oral bacterial detection rates of the matched 2 groups of pregnant women, as shown in Table 5. The differences in detection rates of oral streptococci, lactobacilli, actinomycetes, *Escherichia coli*, *Staphylococcus aureus* and *Pseudomonas aeruginosa* were not statistically significant (*P* > .05). The detection rate of tuberculosis bacilli, Black-pigmented bacteria, and Capnocytophaga in pregnant women with GDM was higher than those in nondiabetic pregnant women (*P* = .000, *P* = .026, and *P* = .030, respectively).

**Table 5:**
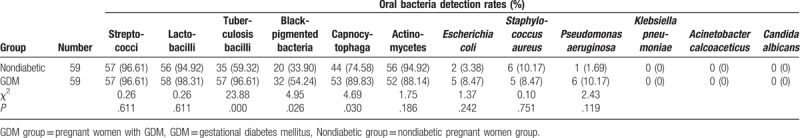
The comparison of oral bacteria detection rates between nondiabetic pregnant women and pregnant women with GDM.

### 3.5 Comparison of the number of oral bacteria

We compared the number of oral bacteria in the matched 2 groups of pregnant women, as shown in Table 6. Compared with nondiabetic pregnant women, pregnant women with GDM had fewer oral streptococci (*P* = .000) and lactobacilli (*P* = .000). Nevertheless, the total number of oral anaerobic bacteria (*P* = .000), tuberculosis bacilli (*P* = .000), Black-pigmented bacteria (*P* = .007), Capnocytophaga (*P* = .000), and actinomycetes (*P* = .000) in pregnant women with GDM were more.

**Table 6:**
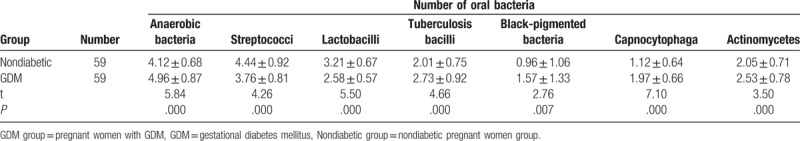
The comparison of number of oral bacteria between nondiabetic pregnant women and pregnant women with GDM.

## 4 Discussion

According to the latest diagnostic criteria recommended by ADA, among the 331 pregnant women included in this study, 65 pregnant women were diagnosed with GDM. The detection rate in this study is 19.6% which is close to the incidence of GDM among pregnant women worldwide (17.8%).^[13]^ A previous study by Getahun et al showed that the incidence of GDM in pregnant women in the United States ranged from 4% to 7%, which was significantly lower than the detection rate of our study.^[14]^ The main reason for this difference is considered to be the inconsistency in the diagnostic criteria of GDM. The diagnostic criteria for GDM used by Getahun et al are the diagnostic criteria recommended by ADA in 1997 and 2000. Specifically, in 100 g OGTT, FBG ≥ 5.3 mmol/L, 1hBG ≥ 10.0 mmol/L, 2hBG ≥ 8.6 mmol/L, 3 hours postprandial BG (3hBG) ≥ 7.8 mmol/L, 2 or more above criteria were met to confirm the diagnosis of GDM.^[15]^ It can be seen that this diagnostic threshold is higher than the new diagnostic criteria recommended by ADA in 2011. The improvement of diagnostic criteria of GDM will help to strictly control BG during pregnancy and improve maternal and neonatal outcomes. However, too strict diagnostic criteria will inevitably increase the detection rate of GDM, increase the psychological pressure of pregnant women, and increase the medical expenses of the state and individuals. Therefore, the diagnostic criteria of GDM should be carefully selected based on the actual situations.

HbA1c is a product of the slow and continuous non-enzymatic reaction of hemoglobin with hexose (mainly glucose) during erythrocyte survival, reflecting the average BG level for nearly 2 to 3 months.^[16]^ Internationally, the use of HbA1c to diagnose and evaluate pre-pregnancy diabetes has been accepted.^[17]^ However, there is no consensus on the role of HbA1c in GDM. In this study, we found that there was a significant correlation between BG levels and HbA1c at various time points in OGTT, suggesting that HbA1c still reflects the average level of BG during pregnancy, which is similar to the results of Rajput et al.^[18]^ However, due to the short course of GDM, the changed hormone levels during pregnancy and the changes of blood volume of pregnant women, HbA1c is not suitable to be an indicator for the diagnosis of GDM.^[19]^ It is only recommended as a supplementary means for detecting BD during pregnancy.

Due to the changes in lifestyle habits and neglect of oral hygiene maintenance, oral flora in pregnant women is often maladjusted that easily leads to periodontal oral infection.^[20]^ Coupled with the gingival vessel dilatation caused by the changes in hormone levels, periodontal infection is likely to further develop into periodontitis.^[21]^ Periodontitis affects not only the mental state but also the appetite of pregnant women. The mental and dietary conditions of pregnant women directly affect the development of the fetus. In addition, studies have found that periodontitis can aggravate the condition of diabetes mellitus in pregnant women with GDM, making BG difficult to control.^[11,22]^

When pregnant women developed GDM, they have more risk factors for oral microbial imbalance. Firstly, the hyperglycemia status of diabetic patients provides abundant nutrition for periodontal bacteria, especially anaerobic bacteria.^[23]^ Secondly, the basal membrane of small blood vessels in the gingival tissues of diabetic patients is thickened, which reduces local blood supply.^[24]^ Thirdly, HbA1c has poor oxygen carrying capacity, which causes local oxygen pressure to drop.^[25]^ Hyperglycemia also increases blood viscosity and platelet aggregation, further aggravating tissue hypoxia.^[26]^ These pathological processes are all beneficial to the growth of periodontal pathogens, supporting our result that the detection rate and the number of oral bacteria in pregnant women with GDM were higher than those in nondiabetic pregnant women.

Since this study is a non-randomized comparative study, there are inevitably some confounding factors. In this regard, we adopted the PSM method to match the factors that may affect the oral health of the 2 groups of pregnant women. In the matched data, there was no significant difference in the age, education level, and BMI at 28 weeks of gestation between the 2 groups. Then, we compared the GI, PI, TMD, PD, and BOP of the matched 59 pregnant women. The results showed that these symptoms associated with periodontitis in pregnant women with GDM are significantly more severe than those in nondiabetic pregnant women, consistent with previous studies.^[10,11]^

In addition, by comparing the oral flora of the matched 2 groups of pregnant women, we found that the detection rate of tuberculosis bacilli, Black-pigmented bacteria, and Capnocytophaga in the mouth of pregnant women with GDM was significantly higher. The oral bacteria of both types of pregnant women are mainly anaerobic bacteria, but the total number of anaerobic bacteria in the mouth of pregnant women with GDM is relatively higher. This suggests that elevated BG in pregnant women with GDM may lead to changes in the type and amount of oral flora, thus breaking the oral microbial balance.

In summary, our study shows that the detection rate and the number of oral bacteria in pregnant women with GDM were higher than those in nondiabetic pregnant women in the second trimester of pregnancy. Pregnant women with GDM should be more alert to periodontitis caused by oral microbial balance. However, because this study was a non-randomized comparative study and eventually included only 59 pairs of pregnant women, clinical study with a larger sample size and more in-depth basic research are still needed to verify our results.

## Author contributions

Conceptualization: Guoyun Wang.

Data curation: Han Yao, Dan Xu.

Formal analysis: Guoyun Wang.

Resources: Guoyun Wang.

Software: Han Yao, Zhichao Zhu.

Validation: Guoyun Wang, Dan Xu, Zhichao Zhu.

Writing-original draft: Han Yao.

**Conceptualization:** Zhichao Zhu, Guoyun Wang.

**Data curation:** Han yao, Dan Xu, Zhichao Zhu.

**Formal analysis:** Han yao, Guoyun Wang.

**Investigation:** Guoyun Wang.

**Methodology:** Han yao.

**Writing – original draft:** Han yao.

**Writing – review & editing:** Dan Xu, Zhichao Zhu, Guoyun Wang.
